# Volatile Compounds as Upcoming Antigiardial Agents: In Vitro Action of Carvacrol, Thymol and p-Cymene on *Giardia lamblia* Trophozoites

**DOI:** 10.3390/pharmaceutics17111380

**Published:** 2025-10-24

**Authors:** Marisa Machado, Ana Silva, Rui Linhares, Carlos Cavaleiro, Maria C. Sousa

**Affiliations:** 1 UCIBIO—Applied Molecular Biosciences Unit, Translational Toxicology Research Laboratory, University Institute of Health Sciences (1H-TOXRUN, IUCS-CESPU), 4585-116 Gandra, Portugal; sonia.marisa@ipsn.cespu.pt; 2 Associate Laboratory i4HB—Institute for Health and Bioeconomy, University Institute of Health Sciences—CESPU, 4585-116 Gandra, Portugal; 3 H^2^M—Health and Human Movement Unit, Instituto Politécnico de Saúde do Norte, Cooperativa de Ensino Superior Politécnico e Universitário (CESPU), CRL, 4760-409 Vila Nova de Famalicão, Portugal; rui.linhares@ipsn.cespu.pt; 4 Unidade Multidisciplinar de Investigação Biomédica (UMIB), Instituto de Ciências Biomédicas Abel Salazar (ICBAS), Universidade do Porto, Rua Jorge Viterbo Ferreira 228, 4050-313 Porto, Portugal; 5 ITR—Laboratory for Integrative and Translational Research in Population Health, Rua das Taipas 135, 4050-600 Porto, Portugal; 6 Faculty of Pharmacy, University of Coimbra, Polo III, Azinhaga de Santa Comba, 3000-548 Coimbra, Portugal; cavaleir@ff.uc.pt (C.C.); mcsousa@ci.uc.pt (M.C.S.); 7 Chemical Process Engineering and Forest Products Research Centre (CIEPQPF), University of Coimbra, Rua Sílvio Lima, 3030-790 Coimbra, Portugal; 8 Center for Neurosciences and Cell Biology (CNC), University of Coimbra, 3004-504 Coimbra, Portugal

**Keywords:** monoterpenes, antiparasitic, antiprotozoal agents, giardiasis, essential oils

## Abstract

**Background/Objectives:** Carvacrol and thymol are monoterpenes present in phenolic-rich essential oils extracted from aromatic plants that exhibit antimicrobial activity. This study evaluates the antiprotozoal effect of carvacrol, thymol and their precursor, p-Cymene, against *Giardia lamblia* and investigates their mechanism of action and cytotoxicity profile. **Methods:** *G. lamblia* susceptibility, cell viability, swelling and adhesion abilities following application of carvacrol, thymol and p-Cymene were assessed. Ultrastructural changes were evaluated using electron microscopy. Cytotoxicity was determined in mammalian cell lines (murine macrophages RAW 264.7 and bovine aortic endothelial cells) exposed to the same IC_50_ concentrations effective against *G. lamblia*. **Results:** Carvacrol and thymol led to significant inhibition of *G. lamblia* trophozoite proliferation (IC_50_ ≅ 50 µg/mL). After 7 h of incubation, total cell number decreased by 30% (*p* < 0.01) with carvacrol and by 50% (*p* < 0.001) with thymol, accompanied by reduced motility and adhesion (<20% attached cells). At IC_50_ concentrations, *G. lamblia* trophozoites exposed to carvacrol and thymol underwent considerable ultrastructural alterations (e.g., aberrant-shaped cells, mitochondrial swelling and autophagosomal structures). Reduced trophozoite motility and adhesion capacity were also observed. In mammalian cells, thymol showed no significant cytotoxicity, whereas carvacrol significantly reduced viability in both cell lines. In contrast, p-Cymene showed no antigiardial activity. **Conclusions:** Our data suggests that carvacrol and thymol disrupt *G. lamblia* trophozoite integrity, possibly through alterations in membrane permeability and osmoregulatory processes. In conclusion, these compounds reveal in vitro antigiardial activity, supporting their potential as antigiardial drugs.

## 1. Introduction

*Giardia lamblia* Kofoid & Christiansen, 1915 (originally described by Lambl in 1859; syn. *Giardia intestinalis*, *Giardia duodenalis* (Stiles, 1902)), is an intestinal flagellated protozoan with global prevalence [1]. This parasite is responsible for giardiasis—an intestinal infection characterized by diarrhea and malabsorption that may lead to nutritional deficiencies and significant morbidity and mortality. *G. lamblia* infects approximately 5.2 individuals per 100,000 for the general population in high-income countries and is especially prevalent among children, being responsible for up to 45% of persistent diarrhea cases in low-income countries [2]. Although there are several therapeutic options, high incidence of failures, frequent relapses, resistances and toxic side effects highlight the need for new antigiardial agents [1].

Plant extracts, due to the diversity and complexity of secondary metabolites, are valuable collections of bioactive compounds, widely used in folk medicine [3,4,5,6]. Essential oils, derived from aromatic plants, offer a rich source of compounds with biological activities, including antimicrobial potential [7,8]. Carvacrol and thymol, abundant in *Thymus vulgaris* L. (Carl Linnaeus, 1753) and *Origanum vulgare* L. (Carl Linnaeus, 1753) species, have demonstrated broad-spectrum antimicrobial activities, including antiparasitic effects reported in protozoan models [9,10,11,12]. Structurally related compounds, such as p-Cymene (a biosynthetic precursor of both carvacrol and thymol), have also shown biological activity, suggesting that these volatile phenolics may offer promising scaffolds for antigiardial drug development [13]. However, their activity against *G. lamblia* remains inadequately studied.

This work investigates the antigiardial effects of carvacrol and thymol and their precursor, p-Cymene, on *G*. *lamblia*, focusing on their mechanism of action and safety profile. We aim to support their potential application as alternative or adjunct therapies against giardiasis.

## 2. Materials and Methods

### 2.1. Chemicals

Carvacrol (98% purity; catalog no. 282197), thymol (≥99% purity; catalog no. 16254), p-Cymene (99% purity; catalog no. C121452), bile bovine (catalog no. B3883), L-cysteine (catalog no. C7352), L-ascorbic acid (catalog no. A4403), ferric ammonium citrate (catalog no. RES20400-A7), RPMI 1640 medium (catalog no. R8758), Dulbecco’s Modified Eagle Medium (DMEM) (catalog no. D6429), dimethyl sulfoxide (DMSO) (catalog no. 472301) and thiazolyl blue tetrazolium bromide (MTT) (catalog no. M5655) were purchased from Sigma-Aldrich (St. Louis, MO, USA). Casitone (catalog no. 225930) and yeast extract (catalog no. 210929) were supplied by Difco Laboratories (now under Gibco/Thermo Fisher Scientific, Detroit, MI, USA), while bovine serum (catalog no. S3113) and the antibiotic solution were obtained from Biochrom K.G. (Berlin, Germany).

### 2.2. Parasites and Cultures

Trophozoites of *G*. *lamblia* (WB strain, ATCC 30957), derived from a clinical isolate associated with chronic diarrhea, were obtained from the American Type Culture Collection (American Type Culture Collection (ATCC), Rockville, MD, USA). The parasites were cultured axenically at 37 °C in 10 mL of Diamond’s TYI-S-33 medium, prepared according to the modifications introduced by Keister [14], and maintained in screw-cap vials. Routine cultures were supplemented with penicillin G and streptomycin sulfate (each at 250 mg/mL). Log-phase trophozoites cultures (2–3 days) were harvested by placing the vials at 4 °C for 15 min to promote detachment, followed by centrifugation at 1500× *g* for 5 min. The resulting pellet was washed twice with phosphate-buffered saline (PBS, pH 7.2), and the number of trophozoites was determined using a Neubauer cell-counter chamber. These trophozoite cultures were then employed to assess the effects of carvacrol and thymol on *G. lamblia* growth, viability, adhesion capacity and structural morphology.

### 2.3. G. lamblia Growth Inhibition Assay

The in vitro susceptibility of *G. lamblia* trophozoites to carvacrol, thymol and p-Cymene was assessed following previously established protocols with minor adaptations [15,16]. Log-phase trophozoites (5 × 10^4^ cells) were incubated for 48 h at 37 °C in fresh culture medium in the presence of increasing concentrations of each compound (ranging from 0.02 to 0.1 mg/mL), using 1.5 mL Eppendorf vials. Control assays were carried out in parallel under identical conditions, but with dimethylsulfoxide (DMSO) alone (at the same final concentration used to dissolve the test compounds), without any active substances. Following incubation, the cultures were cooled at 4 °C for 15 min to promote cell detachment. The number of trophozoites was then quantified using a Neubauer chamber under light microscopy (Nikon Eclipse E100 (Nikon Corporation, Tokyo, Japan)). Results were expressed as total cell count, and the IC_50_ values were calculated based on the dose–response curve.

### 2.4. G. lamblia Viability Assays

Cell viability was assessed through complementary approaches: total cell counts along the time of incubation and morphological evaluation, as previously described [17]. Trophozoites were considered viable when exhibiting a typical pear-shaped morphology, active flagellar motility, preserved ventral disk structure, and light refractivity under optical microscopy. A standard inoculum of 5 × 10^4^ *G. lamblia* trophozoites (WB strain, ATCC 30957) was incubated for 7 h at 37 °C in serum-free Keister-modified medium, supplemented with either carvacrol or thymol at concentrations corresponding to their respective IC_50_ values. Negative controls experiments were carried out under identical conditions using only the vehicle (DMSO). After incubation, trophozoites were counted using a Neubauer hemocytometer, and the results were expressed as a percentage relative to the untreated control. Morphological features were documented using a Nikon Eclipse E400 microscope coupled to a Nikon DN100 Digital Net Camera (Nikon Corporation, Tokyo, Japan).

### 2.5. Measurement of G. lamblia Swelling

To monitor alterations in cell volume, absorbance changes at 540 nm were recorded over time using a Lambda 6 UV/VIS spectrophotometer (Perkin Elmer, Waltham, MA, USA), as previously described [18]. Log-phase *G. lamblia* trophozoites (1.0 × 10^6^ cells) were suspended in 2 mL of reaction medium composed of 200 mM sucrose, 10 mM Tris-Mops (pH 7.4), 1 mM monopotassium phosphate (KH_2_PO_4_) and 10 µM ethylene glycol tetra acetic acid (EGTA), maintained at 37 °C. Carvacrol and thymol were introduced at their respective IC_50_ concentrations. Controls included untreated samples and parallel incubations with 0.1% Triton X-100 to simulate maximal membrane disruption.

### 2.6. Assessment of G. lamblia Adhesion

To investigate whether carvacrol and thymol interfere with trophozoite adhesion, an inoculum of 5 × 10^4^ cells was incubated for 7 h at 37 °C in serum-free medium supplemented with each compound at its respective IC_50_. At the end of the incubation period, both adherent and non-adherent trophozoites were quantified using a Neubauer counting chamber under light microscopy. The adhesion rate was calculated as the proportion of attached cells relative to the total number of trophozoites per replicate.

### 2.7. G. lamblia Transmission and Scanning Electron Microscopy

Morphological changes induced by carvacrol and thymol (at their IC_50_ concentrations) in *G. lamblia* trophozoites were examined using both transmission (TEM) and scanning (SEM) electron microscopy. For TEM analysis, trophozoites were initially fixed in glutaraldehyde buffered with sodium cacodylate, followed by post-fixation in osmium tetroxide and uranyl acetate. Dehydration was performed with ethanol and propylene oxide, as previously described [16]. The samples were then embedded in Epon 812 resin (TAAB 812), and ultrathin sections were prepared and stained with uranyl acetate and lead citrate for structural visualization. SEM samples were processed similarly for fixation and post-fixation, followed by ethanol dehydration, critical point drying with CO_2_ and gold sputter-coating. Ultrastructural observations were performed using a JEOL JEM-100 SX (80 kV) (JEOL Ltd., Tokyo, Japan) for TEM and a JEOL JSM-5400 (15 kV) for SEM (JEOL Ltd., Tokyo, Japan).

### 2.8. Mammalian Cell Cytotoxicity Assay

Cytotoxicity was evaluated in two mammalian cell lines: murine macrophages (RAW 264.7, ATCC TIB-71, from the American Type Culture Collection) and bovine aortic endothelial cells (primary culture). Cells in log phase were detached using trypsin and seeded into 24-well tissue culture plates containing RPMI 1640 medium (macrophages) or DMEM (endothelial cells), each supplemented with 10% fetal bovine serum. Cultures were maintained at 37 °C under microaerophilic conditions until confluence. Subsequently, the medium was replaced by fresh medium containing carvacrol or thymol at their respective IC_50_ concentrations, and cells were incubated for 14 h. Cell viability was evaluated by the tetrazolium-based colorimetric assay (MTT), which detects metabolically active cells capable of reducing the yellow 3-(4,5-dimethylthiazole-2-yl)-2,5 (MTT) to a blue formazan product [19]. After treatment, cells were washed three times with PBS (pH 7.2), followed by addition of 50 μL MTT solution (5 mg/mL in PBS) and 450 μL PBS. After a 1-h incubation at 37 °C, the wells were washed, and formazan crystals were solubilized in 500 μL of DMSO. Absorbance was measured at 530 nm using a microplate reader (Synergy HT, BioTek Instruments, Winooski, VT, USA). Viability was calculated using the formula [(L_2_/L_1_) × 100], where L_1_ is the absorbance of control (untreated) cells and L_2_ is the absorbance of treated cells. Morphological alterations were also monitored by optical microscopy.

### 2.9. Statistical Analysis

All experiments were conducted with three biological replicates, each performed in technical triplicate. Results are expressed as mean values ± standard deviation (SD). The assumption of normality was assessed using the Shapiro–Wilk test. As data followed a normal distribution (*p* > 0.05), statistical comparisons between groups were performed using one-way ANOVA followed by Dunnett’s post hoc test. Differences were considered statistically significant at * *p* < 0.05, ** *p* < 0.01 and *** *p* < 0.001.

## 3. Results

### 3.1. Inhibition of In Vitro Proliferation

**Figure 1:**
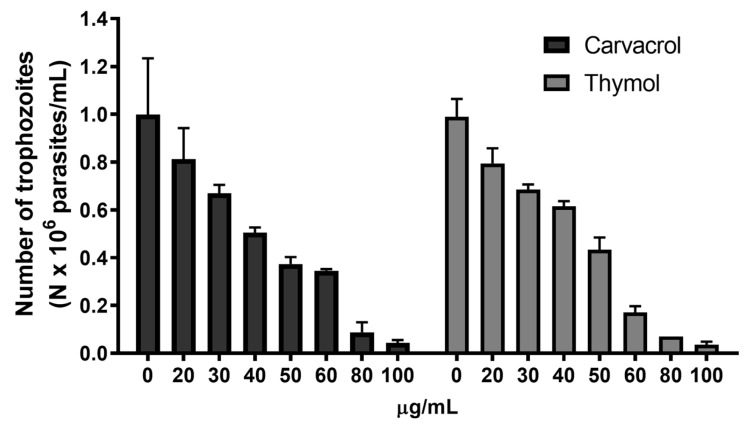
Effects of carvacrol and thymol on *G. lamblia* trophozoite proliferation. Cultures of log-phase trophozoites (5 × 10^4^) were incubated at 37 °C for 48 h as a function of drug concentration.

The impact of increasing concentrations of carvacrol and thymol was assessed in cultured trophozoites to evaluate dose-dependent responses. As shown in Figure 1, carvacrol and thymol revealed a dose-dependent inhibition on *G. lamblia* trophozoite proliferation. At low concentrations (20 µg/mL), both compounds showed slight inhibition (20–30%), while at high concentrations (80 µg/mL), a significant level of inhibition (≥90%) on parasite growth was observed (Figure 1). IC_50_ values were determined as 47 µg/mL for thymol and 51 µg/mL for carvacrol (Table 1). p-Cymene showed no inhibitory effect.

### 3.2. Viability Studies

**Figure 2:**
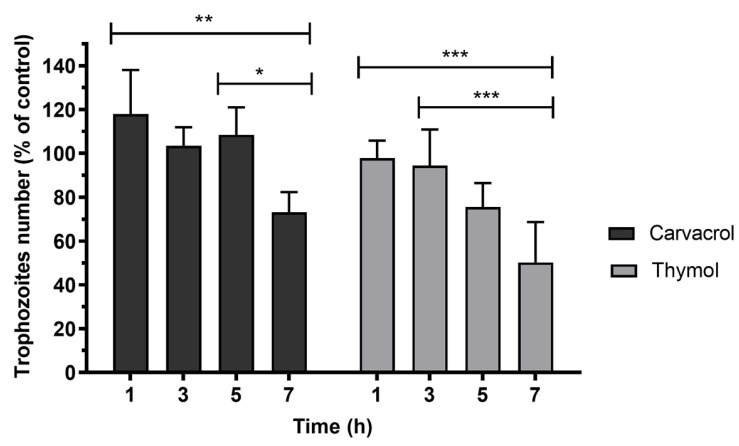
Effects of carvacrol and thymol on *G. lamblia* trophozoite viability. Cell viability of parasites was calculated as a percentage of the control. Determinations were made at 1, 3, 5 and 7 h after incubation with the phenolic compounds at IC_50_ concentrations. Values are expressed as means and SD (n = 6). * *p* < 0.05; ** *p* < 0.01; *** *p* < 0.001.

The time course of *G. lamblia* trophozoite viability was determined using the IC_50_ concentration of carvacrol and thymol, as shown in Figure 2. It was observed that carvacrol did not significantly reduce the cell number after 5 h of incubation. However, after 7 h, carvacrol decreased (*p* < 0.01) the trophozoite number by about 30%. The treatment of cultures with thymol promoted the death of about 25% (*p* < 0.01) of trophozoites after 5 h of incubation. In cultures exposed to thymol for 7 h, cell number decreased to approximately 50% (*p* < 0.001).

**Figure 3:**
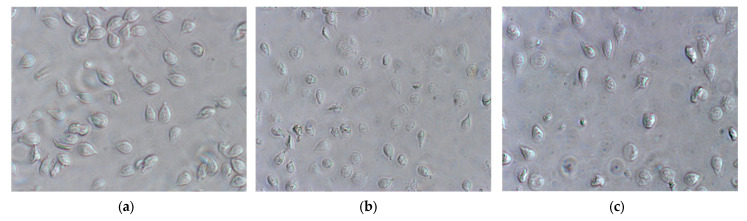
Effects of carvacrol and thymol on G. *lamblia* trophozoite morphology (amplification 400×): (**a**) untreated cells (control), incubated with DMSO (vehicle of phenolic compounds), showing typical morphology; (**b**) cells treated with thymol at IC_50_ and incubated over 7 h; (**c**) cells treated with carvacrol at IC_50_ concentrations over 7 h.

Effects of carvacrol and thymol on viability were also evaluated by morphological criteria. Trophozoites were treated with phenolic compounds at concentrations that inhibited growth at 50% (IC_50_) for 7 h. The untreated G. lamblia trophozoites (Figure 3a) showed a typical pear-shaped body, active flagellar motility, normal ventral disk architecture and dense cytoplasmic content. In contrast, trophozoites exposed to thymol (Figure 3b) and carvacrol (Figure 3c) exhibited significant morphological alterations, including distorted and swollen cell bodies and reduced flagellar movement.

### 3.3. Effects on Cellular Volume

**Figure 4:**
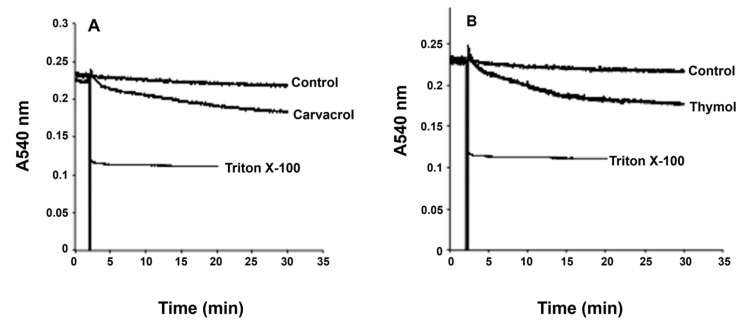
Effects of carvacrol (**A**) and thymol (**B**) on *G. lamblia* trophozoite swelling. Light-scattering measurements at 540 nm show absorbance changes over time, representative of four independent experiments, indicating alterations in cell volume and membrane dynamics.

The antiproliferative effects and morphological changes observed in *G. lamblia* trophozoites following exposure to carvacrol and thymol may be linked to alterations in membrane integrity, either through direct membrane disruption or increased permeability. To help elucidate the underlying mechanism, we assessed changes in trophozoite membrane integrity by monitoring variations in light scattering at 540 nm, which reflect alterations in cell volume and membrane dynamics (Figure 4). The addition of Triton X-100, a known membrane-disrupting agent, led to a marked reduction in light scattering at 540 nm, indicating substantial cellular swelling due to membrane rupture (Figure 4). In contrast, pre-incubation of trophozoite suspensions (10^6^ cells) with carvacrol (Figure 4A) or thymol (Figure 4B) at their respective IC_50_ concentrations did not result in a comparable decrease in absorbance, suggesting that neither compound caused immediate nor extensive membrane disruption under the tested conditions. These results suggest that carvacrol and thymol, at concentrations that inhibited 50% of cell proliferation, did not induce outright cell membrane disruption, but likely induced partial loss of cell integrity and disorganization, sufficient to alter membrane permeability, as shown by the decrease in absorbance (Figure 4).

### 3.4. Adherence Inhibition

**Figure 5:**
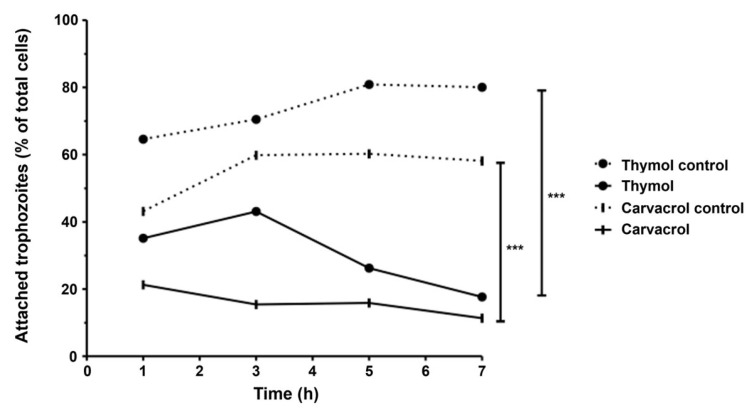
Effects of thymol and carvacrol on *G. lamblia* trophozoite adherence in vitro. Cells were incubated in the presence of phenolic compounds (IC_50_) for 1, 3, 5 and 7 h, and results were expressed as a percentage of *G. lamblia* trophozoites adhered to culture tubes. Error bars indicate standard deviation (SD) of, at least, three replicates; *** *p* < 0.001.

The adhesion capacity of *G. lamblia* trophozoites was evaluated following 1, 3, 5 and 7 h of incubation at 37 °C, using an inoculum of 5 × 10^4^ cells and compound exposure at IC_50_ levels (Figure 5). In control conditions, trophozoite adherence progressively increased over time. However, treatment with carvacrol and thymol led to a time-dependent reduction in adhesion, with significantly fewer cells remaining attached as incubation progressed. In fact, after 7 h of incubation with both thymol and carvacrol, less than 20% of cells were attached (*p* < 0.001). When compared to control assays, thymol promoted a decrease in attachment of about 60% and carvacrol promoted a decrease of 40% (*p* < 0.001).

### 3.5. Ultrastructural Effects

**Figure 6:**
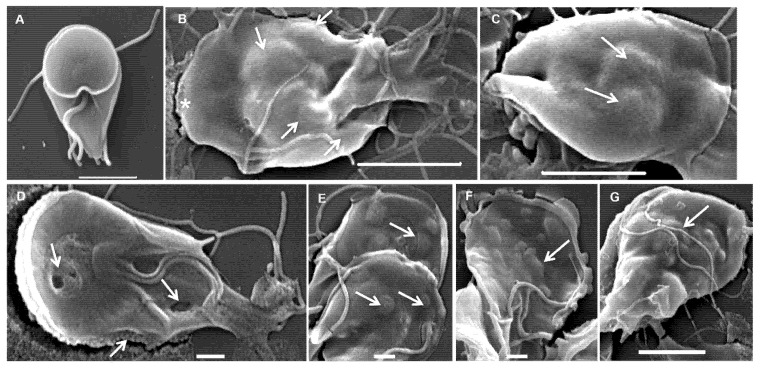
Scanning electron micrographs of *Giardia lamblia* trophozoites exposed to carvacrol (**B**,**C**) and thymol (**D**–**G**): (**A**) untreated cells showing typical pear shape; (**B**–**G**) treated trophozoites showing abnormal shape, irregular ventral and dorsal surface (asterisk) and membrane blebs (arrows). (**A**) Bar = 2 µm; (**B**,**C**,**G**) bars = 5 µm; (**D**–**F**) bars = 1 µm.

**Figure 7:**
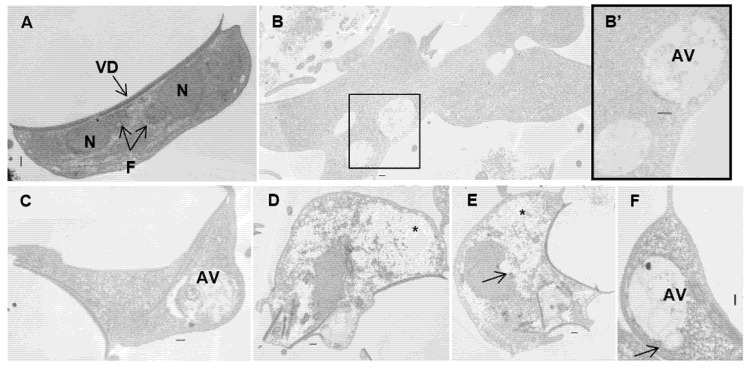
Transmission electron micrographs of *Giardia lamblia* trophozoites exposed to carvacrol (**B**,**B’**) and thymol (**C**–**F**): (**A**) untreated cells showing typical shape; (**B**–**F**) treated trophozoites showing aberrant shape cells, ventral disk and flagella internalization, enlargement of peripherical vesicles and autophagic vesicles, and cytoplasm clearing. Note the electron-dense blocks in the nucleus and cytoplasm (arrows) and empty spaces in the cytoplasm (asterisk). AV—autophagic vesicles; F—axonemal microtubules; N—nucleus; VD—ventral disk. Bars = 2 µm.

To assess ultrastructural alterations, *G. lamblia* trophozoites were incubated for 7 h in the presence or absence of carvacrol and thymol and subsequently examined by scanning (Figure 6) and transmission (Figure 7) electron microscopy. Scanning electron micrographs of untreated cells (Figure 6A) showed a characteristic pear-shaped morphology with a well-defined cytoskeleton comprising four pairs of flagella, a ventral disk, and a ventrolateral flange. In contrast, trophozoites exposed to carvacrol and thymol displayed pronounced morphological disruption, with severely deformed cell surfaces and multiple membrane blebs, resulting in markedly irregular shapes (Figure 6B–G). Also, the rigid structure of the ventral disk appeared to be compromised (Figure 6D).

Transmission electron microscopy of untreated samples revealed the characteristic ultrastructural features of *G. lamblia* trophozoites (Figure 7A). After exposure to carvacrol and thymol, parasite cells became swollen and misshapen, losing the pyriform structure. The main ultrastructural changes observed were the internalization of the ventral disk and flagella (Figure 7D,E), extraction of glycogen granules with cytoplasmic clearing (Figure 7D,E), and compromised integrity of the nuclear envelope (Figure 7E). Additionally, large autophagic vesicles were detected in treated trophozoites (Figure 7B,C,F).

### 3.6. Mammalian Cell Cytotoxicity Assay

The potential cytotoxic effects of carvacrol and thymol were assessed in bovine aortic endothelial cells (primary culture) and RAW 264.7 macrophages using the MTT assay. p-Cymene was not included in these assays due to its lack of antigiardial activity. Carvacrol, at IC_50_ concentrations effective against G. lamblia, significantly reduced cell viability in both tested cell lines. In bovine aortic endothelial cells, the mean difference versus the control was 56.90 (95% CI [32.20–81.60], *p* < 0.0001), while in RAW 264.7 macrophages, the reduction was also significant (mean difference = 33.33, 95% CI [20.15–46.52], *p* < 0.001). Thymol, by contrast, did not cause statistically significant cytotoxic effects in either cell line. In endothelial cells, the mean difference versus the control was 30.20 (95% CI [−0.05–60.45], *p* > 0.05), and in macrophages, the mean difference was 9.80 (95% CI [−4.21–23.81], *p* > 0.05). These results suggest that carvacrol may exert moderate cytotoxic effects at effective concentrations against *G. lamblia*, particularly on endothelial cells.

## 4. Discussion

Giardiasis is a worldwide, highly prevalent intestinal parasitic disease, affecting both developed and developing countries. Current therapeutic options rely mainly on nitroimidazole derivatives, such as metronidazole and tinidazole, which, although effective, present limitations such as undesirable side effects and increasing reports of resistance [1]. This scenario underscores the urgent need for alternative antigiardial agents. Our previous studies found the potential antiprotozoal activity of some essential oils [20,21,22]. Natural products, particularly bioactive compounds from medicinal plants, represent a promising source of novel therapeutics due to their structural diversity and wide range of biological activities. Therefore, the aim of this study was to evaluate the effects of p-Cymene, carvacrol and thymol on *G. lamblia* trophozoites.

Our results revealed that carvacrol and thymol significantly inhibit *G. lamblia* growth at micromolar concentrations (IC_50_ ≅ 50 µg/mL), induce profound ultrastructural changes, and impair parasite adhesion, which is critical for colonization and pathogenesis. *G. lamblia* trophozoite growth inhibition was observed at low concentrations (Figure 1), which is in accordance with previous reports that state a higher susceptibility of protozoans when compared with other microorganisms [23]. In addition, carvacrol and thymol appear to have a favorable safety profile, which makes them potential agents in the management of resistant giardiasis. In fact, a recent phase I clinical trial designed to assess the safety and tolerability of carvacrol capsules in healthy individuals revealed that a one-month treatment with 2 mg/kg/day did not significantly affect metabolic, hematological and electrolyte parameters [24]. Furthermore, these agents seem to have a neuro- and hepatoprotective effect, related to diverse biological properties, including antioxidant, anti-inflammatory and immunomodulatory activities [25,26].

The induction of cell death at IC_50_ concentrations of carvacrol and thymol was minimal during the first three hours of incubation. However, after seven hours, carvacrol and thymol caused approximately 30% (*p* < 0.01) and 50% (*p* < 0.001) of cell death, respectively (Figure 2). These findings suggest that both compounds gradually increased cell membrane permeability, allowing osmotic imbalance and water influx that led to swelling and eventual rupture of the trophozoite membrane. In contrast, prolonged incubation for seven hours caused profound morphological changes, such as distorted cell shapes, swollen bodies, reduced flagellar motility, and cytoplasmic precipitates (Figure 3b,c). Furthermore, pre-incubation with carvacrol or thymol for 30 min did not significantly alter light-scattering measurements compared to Triton X-100 (Figure 4). These results suggest that carvacrol and thymol do not immediately disrupt membrane integrity but instead progressively compromise membrane permeability, leading to growth inhibition. The antimicrobial mechanism of essential oil components is still not fully understood due to the diversity of molecular structures and characteristics. Previous studies indicate that multiple cellular targets may be affected, including components of the cell membrane, electron transport chain, ionic gradients, protein translocation mechanisms, phosphorylation pathways, and other enzyme-dependent processes [27,28,29]. In the case of monoterpenes, their activity seems to be related to the integrity of the cell membrane. Due to their lipophilic nature, these compounds preferentially migrate from the aqueous phase into lipid membranes, promoting membrane expansion and increasing both fluidity and permeability. This interaction with the lipid bilayer compromises membrane integrity, facilitating the loss of cellular homeostasis and ultimately leading to cell dysfunction or death. Such mechanisms have been widely reported for phenolic monoterpenes, which are known to disrupt the ordered structure of biological membranes by integrating into their hydrophobic core and altering key biophysical properties [30,31,32]. Altogether, these findings suggest that the antigiardial activity of carvacrol and thymol may be partially mediated through destabilization of the protozoan plasma membrane, leading to altered membrane properties, subsequent osmotic imbalance, cell swelling, and ultimately cell death (Figure 3 and Figure 4).

However, the hydrophobic nature of essential oil components alone may not fully explain their antimicrobial activity, as certain bacteria (particularly Gram-negative bacteria) appear less susceptible despite having positively charged cell walls [23,33]. Therefore, we hypothesized that these phenolic compounds could inhibit *Giardia* by affecting cytoplasmic metabolic pathways or organelles. In fact, cells exposed to carvacrol and thymol showed cytoplasm precipitates that could be the result of cytoplasmic constituents’ coagulation, such as proteins (Figure 6 and Figure 7), resulting in cytoplasm clearing. At the structural level, *Giardia* trophozoites exhibited marked alterations in size, shape, and internal architecture following exposure to carvacrol and thymol. The morphological changes observed are consistent with a progressive loss of trophozoite viability. Indeed, electron microscopy revealed irregular cell contours and disruption of cytoskeletal organization, including internalization of the ventral disk. This structure plays a decisive role in the attachment of the parasite, a key pathogenesis mechanism [34]. *Giardia* is capable of adhering to both biological and inert surfaces—including various mammalian cell lines, plastic, and glass—which highlights adhesion as a potential target for antigiardial drugs [35,36]. Therefore, we evaluated the ability of carvacrol and thymol to inhibit *G. lamblia* attachment in mammalian cell lines. Our results showed that both compounds severely blocked the attachment of *G. lamblia* (average 40–60% attachment inhibition, and less than 20% of cells remained adhered after seven hours of exposure). A previous study with *Ageratum conyzoides* essential oil reported a similar association between the loss of adhesion to surfaces (through degeneration of the flagella and ventral disks) and parasite death, drawing parallels with the mechanism of action of commercial antigiardial drugs [37]. Recent studies have highlighted the ventral disk as a highly complex and dynamic microtubule-based organelle that is essential for *Giardia’s* attachment to host epithelial surfaces [38]. This structure is not merely a passive suction cup but an intricate architecture composed of microribbons, crossbridges, and lateral crests, which coordinate to generate attachment forces and maintain adhesion under fluid shear stress. Although there is no direct evidence on the effect of carvacrol and thymol on ventral disk dynamics, their known lipophilic properties and membrane-disruptive activities may interfere with microtubule-associated proteins or destabilize lipid–membrane interactions required for disk function. This could partially explain the observed inhibition of *G. lamblia* adhesion, supporting the hypothesis that the ventral disk is a vulnerable target for antigiardial compounds.

In this study, we also observed the formation of autophagosomal vacuoles within the cytoplasm of treated trophozoites. Autophagy is recognized as a cellular survival mechanism activated under stress conditions [39]. The presence of these vesicles may reflect abnormal membrane recycling, suggesting that intense organelle remodeling is occurring, and that this process could be altered or disrupted by the action of carvacrol and thymol.

This study presents some limitations. Firstly, all experiments were conducted in vitro, and the actual bioavailability and pharmacokinetics of carvacrol and thymol in vivo remain unknown. Additionally, the precise molecular targets and pathways involved in their antigiardial action warrant further investigation. A further limitation is the absence of a positive control, such as metronidazole, which would allow a direct comparison with standard antigiardial drugs. Moreover, although cytotoxicity was assessed in mammalian cells, it was only evaluated at the IC_50_ concentrations effective against *G. lamblia*. A full dose–response curve was not created, and thus IC_50_ values for host cells were not determined. As a result, the Selectivity Index (SI) could not be calculated, limiting the assessment of compound selectivity and safety. Nonetheless, the data showed that carvacrol significantly reduced cell viability in both murine macrophages and bovine endothelial cells, whereas thymol did not induce statistically significant cytotoxicity in either cell line. These findings underscore the importance of conducting extended cytotoxicity studies using a broader range of concentrations. Additionally, the mammalian cell lines used in this study do not fully reflect the intestinal environment where *G. lamblia* exerts its pathogenic effects. Therefore, the use of other cell models, such as Caco-2 or HT29-MTX, should be considered in future studies to better simulate host–parasite interactions. Future research should include positive controls, determination of SI values, evaluation of potential synergistic effects with existing antigiardial drugs and exploration of their efficacy in animal models. In addition, further studies are needed to assess the therapeutic potential of carvacrol and thymol, including their bioavailability, cell type-specific interactions, possible synergistic or antagonistic interactions, pharmacokinetics and safety profile in vivo.

## 5. Conclusions

Overall, carvacrol and thymol emerge as promising leads in the development of alternative antigiardial therapies. Further in vivo studies and mechanistic investigations are warranted to validate their potential as safe and effective treatments for giardiasis.

## Figures and Tables

**Table 1:** Inhibitory concentration (IC_50_) of carvacrol, thymol and p-Cymene on *Giardia lamblia*.

Tested Compounds	IC_50_ µg/mL ^1^
Carvacrol	51 (41–63)
Thymol	47 (42–53)
p-Cymene	NA (no activity detected)

^1^ 95% confidence interval. NA—not applicable.

## Data Availability

The raw data supporting the conclusions of this article will be made available by the authors on request.

## Abbreviations

The following abbreviations are used in this manuscript:

DMSO	Dimethylsulfoxide
IC50	Inhibitory Concentration (half-maximal)
SD	Standard Deviation
SEM	Scanning electron microscopy
TEM	Transmission electron microscopy
